# In Vitro Activity of 30 Essential Oils against Bovine Clinical Isolates of *Prototheca zopfii* and *Prototheca blaschkeae*

**DOI:** 10.3390/vetsci5020045

**Published:** 2018-04-24

**Authors:** Simona Nardoni, Francesca Pisseri, Luisa Pistelli, Basma Najar, Mario Luini, Francesca Mancianti

**Affiliations:** 1 Dipartimento di Scienze Veterinarie, Università di Pisa, 56124 Pisa PI, Italy; francesca.mancianti@unipi.it; 2 Scuola Cimi Roma, 00153 Roma RM, Italy; info@francescapisseri.it; 3 Dipartimento di Farmacia, Università di Pisa, 56126 Pisa PI, Italy; luisa.pistelli@unipi.it (L.P.); basmanajar@hotmail.fr (B.N.); 4 Istituto Zooprofilattico Sperimentale della Lombardia e dell’Emilia, Sezione di Lodi, 26900 Lodi LO, Italy; mariovittorio.luini@izsler.it

**Keywords:** *Prototheca zopfii*, *Prototheca blaschkeae*, cattle, mastitis, treatment, essential oils, microdilution

## Abstract

Protothecal mastitis poses an emergent animal health problem in dairy herds, with a high impact on dairy industries, causing heavy economic losses. Current methods of treating protothecal infections are ineffective, and no drug is licensed for use in cattle. The aim of the present study was to check the antialgal activity of 30 chemically defined essential oils (EOs) against *Prototheca zopfii* and *Prototheca blaschkeae* isolated from the milk of dairy cows with mastitis. A microdilution test was carried out to estimate the antialgal effectiveness of the selected chemically defined EOs. The microdilution test showed different degrees of inhibition among the examined *Prototheca* species. The activity of some of the examined EOs seem interesting. In particular, *Citrus paradisi* yielded the lowest minimal inhibitory concentration values (0.75%) for both algal species. *P. zopfii* appeared to be more sensitive to EOs in comparison to *P. blaschkeae*. The present study investigated the in vitro susceptibility of *P. zopfii* and *P. blaschkeae* to a wide range of EOs, obtained from different botanical families. Further investigations are necessary to evaluate the efficacy of EO-based formulations intended for the disinfection of both udder and milking products.

## 1. Introduction

*Prototheca* spp. are achlorophyllous algae, strictly related to *Chlorella*. The genus includes both saprophytic (i.e., *Prototheca stagnora* and *Prototheca ulmea*) and pathogenic (i.e., *Prototheca zopfii*, *Prototheca wickerhami*, *Prototheca blaschkeae*, *Prototheca cutis*, and *Prototheca miyajii*) species [1,2,3,4]. Generally, human and animal protothecoses are considered as rare pathological conditions, apart from the case of bovine mastitis. Bovine protothecosis appears to prevail in contaminated environments where poor milking hygiene exists [5], and is enhanced by the biofilm-producing ability of *P. zopfii*, which may favor its persistence in milking and dairy environments [6,7].

Protothecal mastitis poses an emergent problem in dairy herds [8], and is primarily caused by *P. zopfii* [9] and *P. blaschkeae* [10]. These algae induce severe granulomatous damage to bovine udders, leading to significant irreversible reductions in milk production [11]. This disease has a large negative impact on dairy industries, provoking heavy economic losses. The organoleptic characteristics of milk from infected cows are negatively affected, and thus, these animals should be removed from the herd. Furthermore, *P. zopfii* can be identified as a public health concern, as it is strongly related to increased mortality rates in immunocompromised human patients [12,13].

Current methods of treating protothecal infections are not effective, and *P. zopfii* in particular appears to be resistant to several antimycotic drugs [14,15]. Only amphotericin B appears promising, according to in vitro experiments [16].

Alternative treatments to the conventional antibiotics are welcome. Several natural products, including several plant extracts and essential oils (EOs), have already been examined. A methanolic extract of *Clematis vitalba* was found to be effective against both *P. wickerhami* and *P. zopfii* [17], while a leaf extract of *Camellia sinensis* was active against some strains of *P. wickerhami*, but ineffective against *P. zopfii* [18]. EOs from different botanical species have shown antimicrobial and antifungal actions [19], and can be used as natural preservatives in food [20], or for in vivo treatment of some fungal diseases [21] and prevention of bacterial infections [22]. EOs are multicomponent substances, and their antiseptic activity is often linked to their variety of components, and to their synergistic or antagonistic activity [23]. Furthermore, these natural compounds show anti-biofilm effects [24,25], which renders their use feasible for environmental hygiene.

Data about the sensitivity of *Prototheca* spp. to EOs are scant, and the effectiveness of these substances has only been determined against *P. zopfii* [26] in comparison to *P. wickerhami* [15]. In particular, EOs from *Melaleuca alternifolia* and *Citrus bergamia* [15], and from *Cynnamomum zeylanicum* and *Thymus vulgaris* [26] exerted significant in vitro activity. At the same time, EOs from *Mentha piperita* and *Satureja hortensis* appeared effective in significantly reducing clinical signs of inflammation and fibrosis in vivo, in a murine model of cutaneous protothecosis induced by *P. zopfii* [27].

The aim of the present study was to assess the antialgal activity of 30 chemically defined EOs against *P. zopfii* and *P. blaschkeae* isolated from the milk of dairy cows with mastitis.

## 2. Materials and Methods

EOs from *Pimpinella anisum, Illicium verum*, *Santalum album*, *Helichrysum italicum*, *Rosmarinus officinalis*, *Lavandula hybrida*, *Pelargonium graveolens*, *Salvia sclarea*, *Cynnamomum zeylanicum*, *Foeniculum vulgare*, *Syzygium aromaticum*, *Boswellia sacra*, *Anthemis nobilis*, *Citrus paradisi*, *Citrus bergamia*, *Citrus aurantium*, *Citrus aurantium* var. *dulcis*, *Citrus limon*, *Cymbopogon citratus*, *Ocimum basilicum*, *Origanum majorana*, *Thymus vulgaris*, *Litsea cubeba*, *Origanum vulgare*, *Satureja montana*, *Cistus ladanifer*, *Picea abies*, *Anethum graveolens*, *Thymus capitatus*, and *Myrtus communis* were used for the in vitro assays. All EOs were purchased from Flora s.r.l. (Lorenzana, Pisa, Italy) and were chemically defined. These oils were selected on the basis of the literature (*C. bergamia*, *S. montana*), or their antimycotic properties previously evaluated on different molds and yeasts (i.e., *T. vulgaris*, *O. vulgare*, *L. cubeba*), or their anti-inflammatory, lenitive (i.e., *A. nobilis*, *H. italicum*, *L. hybrida*, *R. officinalis*, *S. sclarea*), and immunostimulating activity (i.e., *C. limon*).

The gas chromatography–mass spectrometry (GC-MS) analysis was performed using a Varian CP-3800 gas chromatograph equipped with a DB-5 capillary column and a Varian Saturn 2000 ion trap mass detector (Varian Inc., Walnut Creek, CA, USA). Analytical conditions and identification of constituents were accomplished according to Pistelli et al. [28]. 

Statistical analysis was performed using the software package Past 3 (version 3.15). The hierarchical cluster analysis (HCA) was performed using Ward’s method, with squared Euclidian distances as a measure of similarity.

*P. zopfii* and *P. blaschkeae* isolated from bovine mastitic milk were used for the in vitro tests. A single isolate for each species was assayed. The organisms were kindly provided by Biobank IZSLER (Brescia, Italy), were characterized by PCR/DNA resolution melting analysis [29], and were maintained onto *Prototheca* Isolation Medium (PIM) at 37 °C. A microdilution test was carried out to estimate the antialgal effectiveness of the selected EOs. The technique was performed using a broth microdilution assay, following the approved standard recommended for yeasts by Clinical and Laboratory Standards Institute M27A_3_ [30], finishing at a 4% dilution. This dilution was chosen as the highest concentration of active that can be administered intramammary [31]. Minimal inhibitory concentration (MIC) was calculated as the lowest dilution at which the algae failed to grow after running the assay in triplicate. Conventional, commonly employed anti-*Prototheca* drugs (i.e., amphotericin B, voriconazole, and posaconazole) were also used. In detail, the in vitro susceptibility of the two algal species was assessed by E-test [32].

## 3. Results

All the identified compounds in the 30 EOs are listed in Table 1, while the main class of constituents are reported in Table 2. The total identified volatile fractions ranged from 86.2% in *S. sclarea* to 100.0% in *C. aurantium*, *C. bergamia*, *C. lemon*, *C. aurantium* var. *dulcis*, *I. verum*, *L. hybrida*, and *S. album*. The composition of all the EOs revealed more than 290 compounds; however, only those with a percentage of 5% or more are reported in Table 1. The main compounds were represented by monoterpene hydrocarbons and oxygenated monoterpenes, while oxygenated sesquiterpenes were abundant only in *S. album* (88.8%). *I. verum*, *P. abies*, and *S. album* showed high percentages of phenylpropanoids (above 90%) which are also the main class of compounds found in *F. vulgare*, although in a lower percentage (56.5%). Non-terpenes were present in high amounts in *A. nobilis* (esters, 75.6%) and *C. zeylanicum* (56.5%).

**Figure 1:**
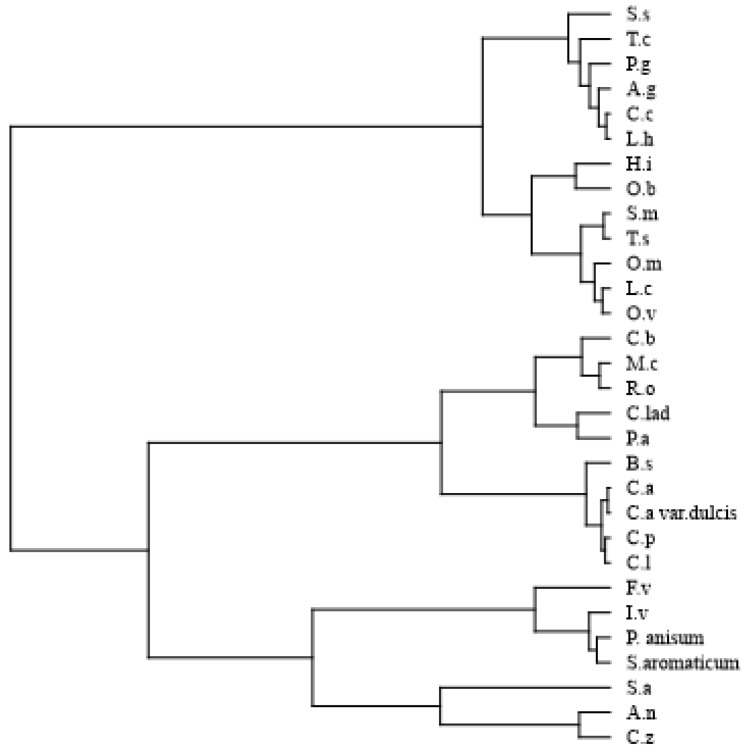
Dendrogram of the hierarchical cluster analysis (HCA) of the 30 tested essential oils (EOs).

Statistical analysis was used to assess the high volume of information provided by the EOs’ composition. The classes of compounds in the EOs were subjected to multivariate analysis. Hierarchical cluster analysis HCA showed the presence of two main groups (Figure 1). The first group (A) includes the following oils: *S. sclarea*, *T. capitatus*, *P. graveolens*, *A. graveolens*, *C. citratus*, *L. hybrida*, *H. italicum*, *O. basilicum*, *S. montana*, *T. vulgaris*, *O. majorana*, *L. cubeba*, and *O. vulgare*. The second group (B) included all remaining oils, and it was further divided into two subgroups: B2 with EOs from *C. zeylanicum*, *A. nobilis*, *S. album*, *S. aromaticum*, *P. anisum*, *I. verum*, and *F. vulgare*, and B1 with the other EOs.

**Figure 2:**
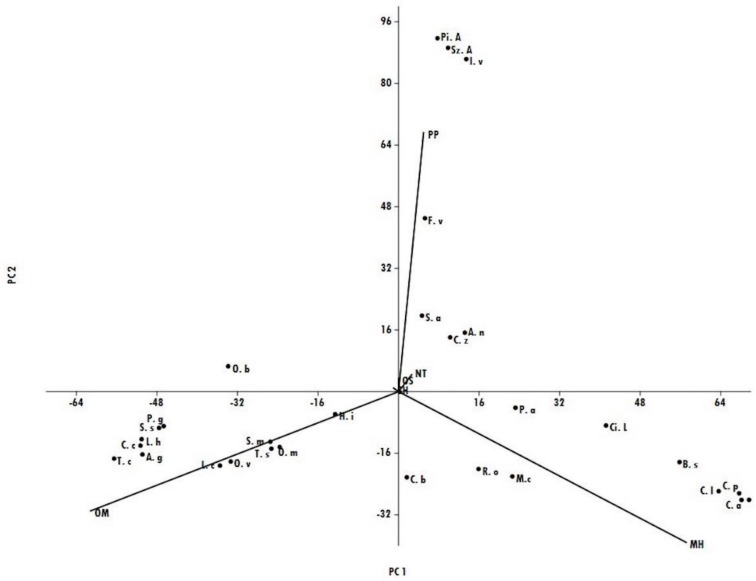
The compound analysis (PCA) plot of the main classes of compounds in the different EOs analyzed.

Principal compound analysis (PCA), where the first axis (PC1) explained for 45.4% and the second axis (PC2) for 33.0% (which resumed 78.4% of the total variability, Figure 2), evidenced that oxygenated monoterpenes (OMs) was the major class of compounds in all the EOs belonging to group A in HCA, and its percentages ranged from 40.6% in *H. italicum* EO to 89.2% in *T. capitatus* EO. The EOs that showed high amounts of phenylpropanoids were located in the upper-right quadrant of the PCA and referred to the plants present in subgroup B2 of the HCA, where *P. anisum*, *I. verum*, and *S. aromaticum* were characterized by the highest percentage of phenylpropanoids (up to 90%), while *C. zeylanicum* and *A. nobilis* oils evidenced relevant amounts of non-terpene derivatives (57.9% and 75.6% respectively). *S. album* EO that belonged to the same subgroup B2 was characterized by the highest percentage of oxygenated sesquiterpenes (88.8%). The presence of monoterpene hydrocarbons was predominant in the EOs of the plants inserted in subgroup B1, especially for all *Citrus* spp. (up to 90%), except for *C. bergamia*, which was characterized by an equivalent amount of monoterpene hydrocarbons and oxygenated monoterpenes.

The microdilution test showed different degrees of inhibition versus the examined *Prototheca* species. *C. paradisi* yielded the lowest MIC values (0.75%) for both algal species. *P. zopfii* appeared to be more sensitive to EOs, in comparison to *P. blaschkeae. T. vulgaris, L. cubeba,* and *O. vulgare* were effective at 0.75% versus *P. zopfii,* and at 1% versus *P. blaschkeae.* More detailed data are reported in Table 3.

## 4. Discussion

The present study is the first to investigate the in vitro susceptibility of *P. zopfii* and *P. blaschkeae* to a wide range of EOs, obtained from different botanical families.

*C. paradisi* appeared to possess the most effective EO against both algal species. The activity of this EO against *Prototheca* spp. had not previously been investigated, and for this reason, our results cannot be compared to already published data.

However, according to comparable results referring to *P. zopfii*’s susceptibility to EOs, MIC values obtained from our microdilution assay seemed to be higher, when compared to those already recorded. In fact, Tortorano et al. [15] reported MICs ranging from 0.03% to 0.12% for *Melaleuca alternifolia*, while MIC values for *C. bergamia* were in agreement with data obtained in the present study.

Grzesiak et al. [26] described *C. zeylanicum* EO as the most effective against *P. zopfii,* followed by *T. vulgaris*, *S. aromaticum*, and *S. sclarea*, with MICs ranging from 0.02% to 0.25%. However, these authors performed the assay following a different method than that implemented in the present study and by Tortorano et al. [15], making a comparison among the different results obtained difficult.

As indicated, *T. vulgaris*, *L. cubeba*, and *O. vulgare* exhibited an inhibitory effect at the level of 0.75% and 1% against *P. zopfii* and *P. blaschkeae*, respectively. 

The main component of *C. paradisi* is limonene. Monoterpenes such as limonene and thymol can exert damage on membrane-embedded enzymes, modify fatty acid composition [33], and affect the respiration and permeability of cell membranes [34]. Furthermore, these monoterpenes are inhibitors of pectin methyl esterase and cellulase, causing consequent damage to fungal cell walls [35]. Limonene, in particular, has also been proven to alter the structure and function of the cell wall in *Saccharomyces cerevisiae* [36].

The activity of some of the examined EOs seem interesting, considering that no drug is currently licensed for use in bovine protothecosis. These compounds could be evaluated for topical administration in a dense excipient for an endomammary treatment of affected cows. The MIC values obtained for the above-mentioned EOs may be employed to treat mammary glands without damaging epithelial cells. Moreover, EOs could be used as disinfectants during milk processing by ensuring a high hygiene level. Protothecal mastitis can in fact be a consequence of poor hygienic conditions, and the search for effective products against these algae is on the rise. The efficacy of some teat disinfectants containing iodine, quaternary ammonium compounds, and dodecylbenzene sulphonic acid has been reported [37], indicating iodine as the most suitable disinfectant. Guanidine is a further compound which is effective both as an antiseptic for human wounds and as a surface disinfectant yielding algicidal activity at low concentration on *P. zopfii* [8]. More recently, the in vitro effect of iodopropynyl butylcarbamate alone and in combination with amphotericin B was assessed on *P. zopfii* and *P. blaschkeae* isolates obtained from dairy herds of different European countries, showing a satisfactory anti-*Prototheca* activity [38].

All of the above-mentioned products are proven to be active versus *P. zopfii*, but to the best of our knowledge only Jagielski et al. [38] provided information on *P. blaschkeae* susceptibility. Although not frequently, this species is also reported as responsible for bovine mastitis outbreaks [39].

Further investigations are needed to evaluate the efficacy of EO-based formulations in the disinfection of both udder and milking products.

## 5. Conclusions

Some among the examined EOs—in particular *C. paradisi* EO—showed interesting antialgal activity against both *P. zopfii* and *P. blaschkeae*. This finding could contribute to broaden the informational outlook on the unconventional treatment of both mastitic cows and dairy environments.

## Figures and Tables

**Table:** (**a**)

Compounds *	Class	LRI ^§^	*A.n*	*A.g*	*B.s*	*C.z*	*C.a*	*C.b*	*C.l*	*C.p*	*C.a.dul*	*C.la*	*C.c*	*F.v*	*H.i*	*I.v*
Propyl butanoate	EST	898	5.5													
α-Thujene	MH	932			54.2	0.3		0.3	0.4				0.1		7.2	
Tricyclene	MH	938			0.2	1.4	0.4	1.1	1.9		0.6		0.2	8.6		1.4
α-Pinene	MH	940	1.2	0.3	6.2					0.5		45.0				
α-Fenchene	MH	951													1.2	
Camphene	MH	955	0.8		0.8	0.6						1.0	1.1	0.3		
Thuja-2.4(10)-diene	MH	959			7.3											
β-Pinene	MH	981	0.2		1.1	0.5	0.1	5.4	11.9	0.1		0.6		1.0	1.0	
α-Terpinene	MH	1019			5.1	1.0			0.2			0.1			0.1	0.2
*p*-Cymene	MH	1028		3.2		3.0		0.1	0.2			6.1		1.9	1.1	0.1
Limonene	MH	1032	0.7	3.2	0.4		94.7	33.2	65.7	92.2	95.5	1.8	2.0	6.5	7.0	3.9
β-Phellandrene	MH	1033				5.9										
1.8-Cineole	OM	1036											0.3		2.3	
Isobutyl angelate	EST	1053	34.5													
γ-Terpinene	MH	1062			0.1			6.4	9.3					0.3	0.3	0.1
Artemisia ketone	MH	1065	7.4													
Fenchone	OM	1090												20.1		
*trans*-Sabinene hydrate	OM	1101							0.2							
Linalool	OM	1102			0.2	6.3	0.4	14.2		0.2	0.5	0.5	1.5	0.4	0.8	0.2
Camphor	OM	1148												0.5		
Isoamyl angelate	EST	1162	18.7													
Propyl tiglate	EST	1166	5.3													
4-Terpineol	OM	1180				0.3									0.2	0.3
3.9-Epoxy-p-menth-1-ene	OM	1186		12.0												
Menthyl chavicol	PP	1198												8.6		0.3
Citronellol	OM	1231														
Neral	OM	1242	0.1					0.4	0.7				35.2			
Carvone	OM	1248		70.4												
Geraniol	OM	1259											4.4			
Linalyl acetate	OM	1260	0.5				1.4	31.7								
(E)-Cinnamaldehyde	NT	1274				56.4										
Geranial	OM	1276						0.4	1.2		0.1		38.4			
Citronellyl formate	OM	1280														
*iso*-Bornyl acetate	OM	1287														
(E)-Anethol	PP	1290		0.3										46.9		89.8
Carvacrol	OM	1301														
Thymol	OM	1307										0.3				
α-Limonene diepoxide	OM	1347														
Eugenol	PP	1361				3.0										
Neryl acetate	OM	1368						0.8	0.7						31.8	
β-Caryophyllene	SH	1418				10.3	0.2	0.7	0.4	0.7		0.2	2.3		3.1	0.3
Neryl propanoate	OM	1454													5.1	
*ar*-Curcumene	SH	1484													5.6	
Eugenol acetate	PP	1529														
5-*epi*-7-*epi*-*α*-Eudesmol	OS	1606													5.5	
γ-Eudesmol	OS	1634													0.5	
T-Cadinol	OS	1642														
β-Eudesmol	OS	1649													0.2	
Valerianol	OS	1655													0.3	
7-*epi*-α-Eudesmol	OS	1664														
(Z)-α-Santalol	OS	1675														
(Z)-β-*trans*-Santalol	OS	1710														
Unknown			7.9	2.8	5.1	0.3				0.8		6.2	2.4	0.3	5.8	
TOTAL			92.1	97.2	94.9	99.7	100.0	100.0	100.0	99.2	100.0	93.9	97.6	99.7	94.3	100.0

* Single compounds that showed a percentage less than 5% in at least one of the tested EOs are removed from this table. ^§^ Linear retention index. ***A.n*** (*Anthemis nobilis*); ***A.g*** (*Anethum graveolens*); ***B.s*** (*Boswellia sacra*); ***C.z*** (*Cynnamomum zeylanicum*); ***C.a*** (*Citrus aurantium*); ***C.b*** (*Citrus bergamia*); ***C.l*** (*Citrus limon*); ***C.p*** (*Citrus paradisi*); ***C.a.dul*** (*Citrus aurantium* var. *dulcis*); ***C.la*** *(Cistus ladanifer*); ***C.c*** (*Cymbopogon citratus*); ***F.v*** *(Foeniculum vulgare*); ***H.i*** (*Helichrysum italicum*); ***I.v*** (*Illicium verum*). MH (Monoterpene Hydrocarbons), OM (Oxygenated Monoterpenes), SH (Sesquiterpene Hydrocarbons), OS (Oxygenated Sesquiterpenes), DH (Diterpene Hydrocarbons), OD (Oxygenated Diterpenes), PP (Phenylpropanoids), NT (Non Terpenes), EST (Esters).

**Table:** (**b**)

Compounds *	Class	LRI ^§^	*L.h*	*L.c*	*M.c*	*O.b*	*O.m*	*O.v*	*P.g*	*P.ab*	*P.a*	*R.o*	*S.s*	*S.al*	*S.m*	*S.a*	*T.c*	*T.v*
Propyl butanoate	EST	898																
α-Thujene	MH	932			0.6		0.8	0.8		0.5		0.2			0.3			*0.1*
Tricyclene	MH	938	0.5	1.5	49.0							0.2						
α-Pinene	MH	940				0.2	0.7	1.0		10.8	0.1	37.9			0.5			*0.9*
α-Fenchene	MH	951						0.1										
Camphene	MH	955	0.5	0.3	0.2	0.1				4.6		5.4			0.3			0.3
Thuja-2.4(10)-diene	MH	959																
β-Pinene	MH	981	0.4	1.2	0.9	0.5	0.7	0.4		1.5		5.0			0.9			
α-Terpinene	MH	1019					4.7	2.1				0.3			1.2		0.3	0.8
*p*-Cymene	MH	1028		0.2	2.7		4.2	9.3							9.0		2.4	15.3
Limonene	MH	1032		16.3	5.9	0.3	2.1	0.7		20.4		3.3						0.4
β-Phellandrene	MH	1033																
1.8-Cineole	OM	1036	7.7	2.3	29.0	5.9	0.1	0.8				22.0			1.0		1.3	0.7
Isobutyl angelate	EST	1053																
γ-Terpinene	MH	1062	0.1	0.1			7.9	5.3				0.5			6.1		1.6	2.9
Artemisia ketone	MH	1065																
Fenchone	OM	1090																
*trans*-Sabinene hydrate	OM	1101					12.8	1.8										3.8
Linalool	OM	1102	31.5	1.5	1.5	46.0			3.9		3.5	0.6	8.1		3.1		2.3	
Camphor	OM	1148	7.3			0.8	0.2					7.6			0.7			0.5
Isoamyl angelate	EST	1162																
Propyl tiglate	EST	1166																
4-Terpineol	OM	1180	4.0	0.1	0.2	0.3	17.6	0.9				0.3			0.7		1.7	2.4
3.9-Epoxy-p-menth-1-ene	OM	1186																
Menthyl chavicol	PP	1198				1.1					0.4							
Citronellol	OM	1231							44.5									
Neral	OM	1242		32.5					0.2									
Carvone	OM	1248					0.6			0.8					1.9			
Geraniol	OM	1259		0.5		0.2	2.7		13.7									
Linalyl acetate	OM	1260	26.8				3.2						54.7		1.2			
(E)-Cinnamaldehyde	NT	1274																
Geranial	OM	1276		36.4					0.7									
Citronellyl formate	OM	1280							7.3									
*iso*-Bornyl acetate	OM	1287				1.6	0.2	0.1		8.9		3.3	0.1					
(E)-Anethol	PP	1290									94.6							
Carvacrol	OM	1301					20.8	65.9							47.1		82.5	0.2
Thymol	OM	1307					0.2	0.9							2.6			52.6
α-Limonene diepoxide	OM	1347											8.6					
Eugenol	PP	1361				11.5										77.9		
Neryl acetate	OM	1368	0.4										0.1					
β-Caryophyllene	SH	1418	2.2	0.8	0.5	0.3	1.7	3.7	0.7	4.4		4.1			3.6	8.9	3.0	6.8
Neryl propanoate	OM	1454																
*ar*-Curcumene	SH	1484													0.3			
Eugenol acetate	PP	1529														12.2		
5-*epi*-7-*epi*-*α*-Eudesmol	OS	1606																
γ-Eudesmol	OS	1634												5.2				
T-Cadinol	OS	1642	0.2			5.8									0.2			
β-Eudesmol	OS	1649												5.5				
Valerianol	OS	1655												14.4				
7-*epi*-α-Eudesmol	OS	1664												5.9				
(Z)-α-Santalol	OS	1675												27.1				
(Z)-β-*trans*-Santalol	OS	1710												10.8				
Unknown			6.2	0.8	2.0	1.8	0.0	1.7	1.4	5.1	13.9	5.6	5.4		1.3	1.7		
TOTAL			100.0	99.4	93.9	99.2	98.1	98.2	99.3	98.3	98.6	94.9	86.2	94.4	94.6	100.0	98.7	97.3

Single compounds that showed a percentage of less than 5% in at least one of the tested EOs are removed from this table. ^§^ Linear retention index. ***L.h** (Lavandula hybrida); **L.c** (Litsea cubeba*); ***M.c*** *(Myrtus communis*); ***O.b** (Ocimum basilicum*); ***O.m*** *(Origanum majorana*); ***O.v*** *(Origanum vulgare*); ***P.g** (Pelargonium graveolens*); ***P.ab*** *(Picea abies*); ***P.a*** *(Pimpinella anisum*); ***R.o*** *(Rosmarinus officinalis*); ***S.s*** *(Salvia sclarea*); ***S.al*** *(Santalum album*); ***S.m*** *(Satureja montana*); ***S.a*** *(Syzygium aromaticum*); ***T.c*** *(Thymus capitatus*); ***T.v*** *(Thymus vulgaris*). MH (Monoterpene Hydrocarbons), OM (Oxygenated Monoterpenes), SH (Sesquiterpene Hydrocarbons), OS (Oxygenated Sesquiterpenes), DH (Diterpene Hydrocarbons), OD (Oxygenated Diterpenes), PP (Phenylpropanoids), NT (Non Terpenes), EST (Esters).

**Table:** (**a**)

Class of Compounds *	*A.n*	*A.g*	*B.s*	*C.z*	*C.a*	*C.b*	*C.l*	*C.p*	*C.a.dul*	*C.la*	*C.c*	*F.v*	*H.i*	*I.v*
Monoterpene hydrocarbons (MH)	10.3	10.1	84.3	15.5	97.4	49.0	94.3	96.2	98.7	57.0	3.9	22.1	19.2	7.3
Oxygenated monoterpenes (OM)	5.9	85.9	6.7	7.4	1.9	48.5	3.6	0.5	0.6	16.6	86.3	21.1	40.6	0.7
Sesquiterpene hydrocarbons (SH)	0.2		3.7	14.7	0.2	2.5	2.0	1.9		11.0	4.5		22.5	1.1
Oxygenated sesquiterpenes (OS)	0.2			0.8						6.6	0.9		8.9	0.1
Diterpene hydrocarbons (DH)														
Oxygenated diterpenes (OD)														
Phenylpropanoids (PP)		0.3	0.2	3.4								56.5		90.8
Non-terpenes (NT)		0.9		57.9	0.5	0.1	0.1	0.6	0.7	2.7	2.0			
Esters (EST)	75.6												3.1	
TOTAL Identified	92.1	97.2	94.9	99.7	100.0	100.1	100.0	99.2	100.0	93.9	97.6	99.7	94.3	100.0

* Each class of compounds represent the total amount (%) of the identified constituents. ***A.n*** (*Anthemis nobilis*); ***A.g*** (*Anethum graveolens*); ***B.s*** (*Boswellia sacra*); ***C.z*** (*Cynnamomum zeylanicum*); ***C.a*** (*Citrus aurantium*); ***C.b*** (*Citrus bergamia*); ***C.l*** (*Citrus limon*); ***C.p*** (*Citrus paradisi*); ***C.a.dul*** (*Citrus aurantium* var. *dulcis*); ***C.la*** (*Cistus ladanifer*); ***C.c*** (*Cymbopogon citratus*); ***F.v*** (*Foeniculum vulgare*); ***H.i*** (*Helichrysum italicum*); ***I.v*** (*Illicium verum*).

**Table:** (**b**)

Class of Compounds *	*L.h*	*L.c*	*M.c*	*O.b*	*O.m*	*O.v*	*P.g*	*P.ab*	*P.a*	*R.o*	*S.s*	*S.al*	*S.m*	*S.a*	*T.c*	*T.v*
Monoterpene hydrocarbons (MH)	6.4	21.3	61.2	2.3	27.7	22.4		38.2	0.1	56.5			19.6		4.6	21.5
Oxygenated monoterpenes (OM)	85.0	75.7	31.1	56.1	66.6	71.2	83.4	23.1	3.5	36.8	78.2	0.6	61.9		89.2	64.1
Sesquiterpene hydrocarbons (SH)	5.4	0.9	1.2	20.0	3.2	4.2	7.8	25.0		4.4	0.9	5.1	11.9	9.5	3.8	9.2
Oxygenated sesquiterpenes (OS)	1.3			7.9	0.4	0.4	6.9	12.0		0.3	4.9	88.8	1.1	0.4	0.7	
Diterpene hydrocarbons (DH)															0.2	
Oxygenated diterpenes (OD)											1.3					
Phenylpropanoids (PP)				12.7			1.2		95.0					90.1		
Non-terpenes (NT)	0.8	1.5			0.1						0.2				0.3	0.8
Esters (EST)	1.1		0.3	0.2												1.7
TOTAL Identified	100.0	99.4	93.9	99.2	98.1	98.2	99.3	98.3	98.6	94.9	86.2	94.4	94.6	100.0	98.7	97.3

* Each class of compounds represent the total amount (%) of the identified constituents. ***L.h*** (*Lavandula hybrida*); ***L.c*** (*Litsea cubeba*); ***M.c*** (*Myrtus communis*); ***O.b*** (*Ocimum basilicum*); ***O.m*** (*Origanum majorana*); ***O.v*** (*Origanum vulgare*); ***P.g*** (*Pelargonium graveolens*); ***P.ab*** (*Picea abies*); ***P.a*** (*Pimpinella anisum*); ***R.o*** (*Rosmarinus officinalis*); ***S.s*** (*Salvia sclarea*); ***S.al*** (*Santalum album*); ***S.m*** (*Satureja montana*); ***S.a*** (*Syzygium aromaticum*); ***T.c*** (*Thymus capitatus*); ***T.v*** (*Thymus vulgaris*).

**Table 3:** Minimal inhibitory concentration (MIC) of tested EOs and conventional antifungal drugs.

EOs	*Prototheca zopfii*	*Prototheca blaschkeae*
	MIC (%)	
*Pimpinella anisum*	4	>4
*Illicium verum*	>4	>4
*Santalum album*	>4	>4
*Helichrysum italicum*	>4	>4
*Rosmarinus officinalis*	>4	>4
*Lavandula hybrida*	>4	>4
*Pelargonium graveolens*	>4	>4
*Salvia sclarea*	>4	>4
*Cynnamomum zeylanicum*	>4	>4
*Foeniculum vulgare*	>4	>4
*Syzygium aromaticum*	>4	>4
*Boswellia sacra*	>4	>4
*Anthemis nobilis*	>4	>4
*Citrus paradisi*	0.75	0.75
*Citrus bergamia*	2	0.75
*Citrus aurantium*	>4	>4
*Citrus aurantium* var. *dulcis*	>4	>4
*Citrus limon*	>4	>4
*Cymbopogon citratus*	1	>4
*Ocimum basilicum*	1	>4
*Origanum majorana*	1	>4
*Thymus vulgaris*	0.75	1
*Litsea cubeba*	0.75	1
*Origanum vulgare*	0.75	1
*Satureja montana*	4	4
*Cistus ladanifer*	4	4
*Picea abies*	4	4
*Anethum graveolens*	4	4
*Thymus capitatus*	4	4
*Myrtus communis*	4	4
**Conventional drugs**	MIC (μg/mL)	
Posaconazole	0.38	0.5
Voriconazole	6	6
Amphotericin B	0.25	0.19

Both species were susceptible to amphotericin B and posaconazole, while not sensitive to voriconazole.
